# Manual of Human Microscopic Anatomy

**Published:** 1855-08

**Authors:** 


Manual of Human Microscopic Anatomy. By A. Koleker, Professor of Anatomy and Physiology in Wurzburg. Translated by George Busk, F. R. S., and Thomas Huxley, F. R. S. Edited, with notes and additions, by J. Da Costa, M. D. Illustrated by three hundred and thirteen engravings on wood. Philadelphia—Lippincott, Grambo, & Co., 1854.

The American Edition of Professor Koleker's "Manual of Human Histology," contains all that is known in this department of science up to the date of its publication. So far as medicine is concerned, a knowledge of the minute structure of parts is quite as necessary for the perfect understanding of the pathology of disease as the coarse anatomy of the body, and indeed still more so; for the one only enables us to learn, so to speak, its geographical position, while the other reveals to us the morphological elements of the structure which it invades, and the changes which it induces.

With the invention of the microscope and its application to the study of natural history, was ushered in a new era in the annals of our science. A host of observers immediately commenced the interesting study of the new world which had been revealed by this wonderful instrument. Amid so much that was new and startling, it was not strange that the imaginations of many were excited, and that the facts which they saw were mingled with vague theories and fanciful interpretations. To Schleiden and Sehwann we are indebted for the foundations of microscopy as a science; while among those who have been most zealous in promoting that science, in correcting errors, and bringing to light new truths, the author of this work deservedly holds the most prominent position. The translators and the American editor have each done their duty, and the notes which they have appended add not a little to the value of the work.

For sale by D. B. Cooke & Co. J.

